# A multi-centre randomized controlled trial comparing arthroscopic osteochondroplasty and lavage with arthroscopic lavage alone on patient important outcomes and quality of life in the treatment of young adult (18–50) Femoroacetabular impingement

**DOI:** 10.1186/s12891-015-0500-y

**Published:** 2015-03-20

**Authors:** FIRST Investigators

**Affiliations:** Department of Surgery, Division of Orthopaedic Surgery, McMaster University, 1200 Main St West, 4E15, Hamilton, ON L8N 3Z5 Canada

**Keywords:** Femoroacetabular impingement, Arthroplasty, Osteochondroplasty, Lavage, Randomized controlled trial, Protocol

## Abstract

**Background:**

Several cross-sectional studies have estimated that the prevalence of femoroacetabular impingement (FAI) ranges from 14-17% among asymptomatic young adults to almost 95% among competitive athletes. With FAI, there is abnormal contact between the proximal femur and the acetabulum, resulting in abnormal mechanics with terminal motion such as hip flexion and rotation. This condition results from bony anomalies of the acetabular rim (Pincer) and or femoral head/neck junction (CAM) and typically causes hip pain and decreased hip function. The development of hip pain potentially serves as an indicator for early cartilage and labral damage that may result in hip osteoarthritis. Although surgical correction of the misshaped bony anatomy and associated intra-articular soft tissue damage of the hip is thought to improve hip pain and alter the natural history of degenerative disease, the supportive evidence is based upon low quality observational studies. The Femoroacetabular Impingement RandomiSed controlled Trial (FIRST) compares outcomes following surgical correction of the impingement morphology (arthroscopic osteochondroplasty) with/without labral repair versus arthroscopic lavage of the hip joint in adults aged 18 to 50 diagnosed with FAI.

**Methods and design:**

FIRST is a multi-centre, randomized controlled trial with a sample size of 220 patients. Exclusion criteria include the presence of hip syndromes, previous surgery or trauma to the affected hip, and significant medical comorbidities. The primary outcome is pain and the secondary outcomes include patient function, quality of life, complications, and cost-effectiveness – all within one year of follow-up. Patients are stratified based on centre and impingement sub-type. Patients, outcome assessors, data analysts, and the Steering Committee are blinded to surgical allocation. Using an intention-to-treat approach, outcome analyses will be performed using an analysis of covariance and descriptive statistics.

**Discussion:**

Symptomatic FAI is associated with chronic hip pain, functional limitations, and secondary osteoarthritis. Therefore, optimizing treatment has the potential to improve the lives millions of young, active persons who are diagnosed with this condition. Few orthopaedic surgical trials have similar potential to shift the paradigm of care dramatically towards (or away) from surgical bony and soft tissue interventions.

**Trial registration:**

The FIRST trial is registered with clinicaltrials.gov (NCT01623843).

## Background

**Figure 1:**
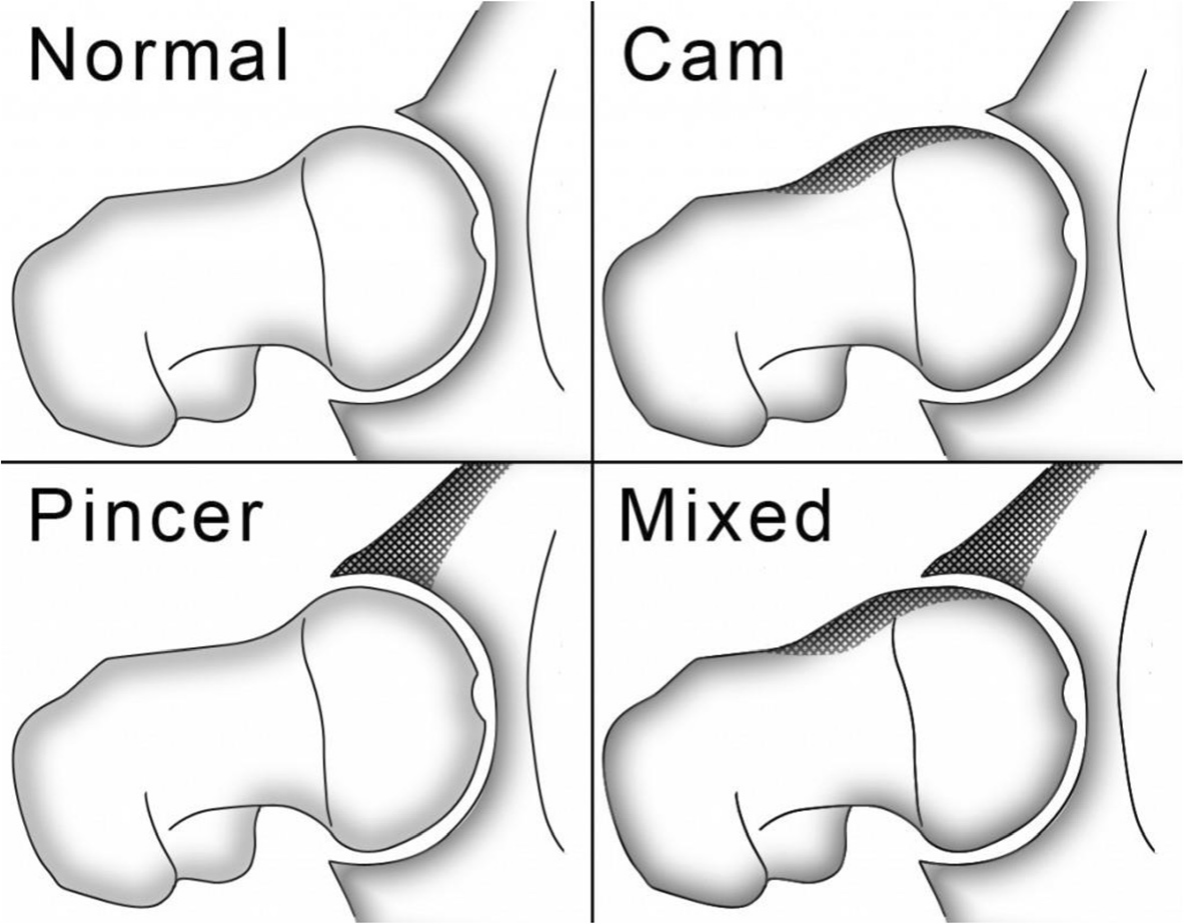
**Femoroacetabular impingement sub-types (from** http://www.kevinneeld.com/2011/training-around-femoroacetabular-impingement **).**

Femoroacetabular impingement (FAI) is a recently described condition that causes hip pain in the young adult. Some cross-sectional studies have estimated that the prevalence of hip impingement ranges from 14-17% among asymptomatic young adults to almost 95% among competitive athletes [1-3]. FAI occurs as a result of a size and shape mismatch between the femoral head (ball) and the acetabulum (socket). FAI is typically classified into two subtypes; CAM type (a misshaped femoral head) or Pincer type (an over covered or deep socket) (Figure 1). Most patients have a combination of both types of impingement (Mixed) [4]. With FAI, the abnormal femoral head-neck junction and acetabular rim of the hip joint collide or “impinge” during movements such as hip flexion and rotation [5]. Typically, patients with this condition may experience hip pain and loss of hip function. The development of hip pain in this manner is believed to result in early cartilage and labral damage, potentially resulting in hip osteoarthritis [5].

Although no definitive long term longitudinal studies exist, the current literature is suggestive of a relationship between longstanding FAI and hip osteoarthritis [6]. Agricola and colleagues found that an alpha angle greater than 83 degrees (angle greater than 50 is used to describe CAM impingement) resulted in an odds ratio 9.66 for the development of hip osteoarthritis within 5 years follow-up [7]. Clohisy et al. also found that approximately one third of patients with hip joint failure secondary to osteoarthritis had deformities related to FAI [8].

This association has fuelled a more aggressive approach to treating the painful and dysfunctional hip with surgical treatment to correct abnormalities of FAI. Colvin et al. reported an 18-fold increase in hip arthroscopic procedures between 1999 and 2009 amongst orthopaedic graduates [9]. Likewise, Bozic et al. demonstrated a 600% increase in hip arthroscopy amongst orthopaedic surgeons completing their Board examinations in the United States (US) from 2006 to 2010 [10]. More recently, estimates suggest that the rate of hip arthroscopy procedures will double over a 5 year span from 30,000 in 2008 to over 70,000 in 2014 in the US alone [11]. Increasingly, academic institutions in the US, Canada, and worldwide are employing orthopaedic surgeons who have expertise in hip impingement surgery, and the publicity from popular athletes having hip surgery for FAI continues to drive the mass demand for surgical intervention [12]. A corresponding dramatic increase in health care costs is expected for these procedures.

### Lack of compelling clinical evidence

Patients with FAI often fail non-operative interventions, including rest, physical therapy, oral anti-inflammatories, and hip injections [13]. Surgical intervention usually involves correcting the existing deformities by reshaping the ball and socket (“osteoplasty” or “rim trimming”) so that they fit together more easily while repairing any other existing soft tissue damage in the hip joint (e.g. labral repair). FAI is managed with open surgical techniques (surgical hip dislocation) as well as keyhole or arthroscopic surgical techniques. Although several reviews have shown no significant differences in clinical outcome with the use of either technique, the arthroscopic approach is becoming increasingly popular due to its minimally invasive technology and out-patient basis of the surgery. Although correction of the misshaped bony anatomy and associated intra-articular soft tissue damage of the hip is thought to appease impingement and improve pain and function, the evidence is based upon observational studies.

We conducted several systematic reviews of the available literature to identify optimal treatment strategies [14-19]. In a systematic review of two electronic databases (MEDLINE and EMBASE), we identified 298 relevant studies on the topic of FAI between 2005 and 2010. Over this 5 year period, there was an approximate five-fold increase in the number of FAI related publications. The majority of publications consisted of case series with a notable absence of clinical trials [17].

In another review of the outcomes of surgical deformity correction (osteoplasty), of 1,103 potentially relevant studies, 14 fulfilled our eligibility criteria. All studies were observational designs (12 case series, 1 prospective cohort, and 1 case–control study). Correction resulted in significant improvements in patients’ hip pain and function [15]. In another review, we evaluated the impact of labral repairs on patient outcomes [19]. We identified six eligible observational studies (5 retrospective comparative designs, 1 prospective cohort) involving 490 patients. Four studies reported that labral repair had greater postoperative improvements in functional scores compared to labral debridement alone. Five studies reported statistically significant improvements with labral repair [19].

In a survey of 200 surgeon members of the Canadian Orthopaedic Association who treat young adults with hip pain, considerable disparity in preferences for surgical treatment of FAI existed, with 50% endorsing both bony and soft tissue procedure, 2% soft tissue only, 10% bony procedures only, and 24% unsure about any surgical option. In contrast, surgeons achieved near consensus (90%) that there is a need for a well conducted randomized controlled trial (RCT) to evaluate the efficacy of current surgical interventions [20].

The significant benefits in outcome purported to arise from surgical management of FAI (osteochondroplasty) are solely driven by observational studies, often with no controls. Given the growing awareness of the evidence gap and lack of clear consensus on the utility of FAI surgery fuelled the design and start-up activities of the Femoroacetabular Impingement RandomiSed controlled Trial (FIRST) - a multicenter RCT to evaluate operative management of FAI. There is a critical need to confirm, or refute, this data with a RCT to overcome the biases associated with lack of randomization, lack of concealment, lack of blinding and lack of independence in outcome assessment.

### Study objectives

The primary research objective is to assess whether surgical correction of the impingement morphology (arthroscopic osteochondroplasty) with/without labral repair, in adults aged 18 to 50 diagnosed with FAI, provides decreased pain at 12 months compared to arthroscopic lavage of the hip joint as measured by a Visual Analog Scale (VAS) [21-23].

The secondary research objectives are to assess whether arthroscopic osteochondroplasty with/without labral repair, in adults aged 18 to 50 diagnosed with FAI, provides improved patient outcomes at 12 months compared to arthroscopic lavage of the hip joint including:

1. Functional outcomes as measured by the Hip Outcome Score (HOS) [24]
2. Generic physical and mental health as measured by the Short Form-12 (SF-12) [25]
3. Impact of hip-specific disease on function and lifestyle in the young, active patient as measured by the International Hip Outcome Tool (iHOT-12) [26]
4. Health utility as measured by the EuroQol (EQ-5D) [27]
5. Urinary and sexual function as measured by the International Consultation on Incontinence Questionnaire - Male/Female Lower Urinary Tract Symptoms (ICIQ-MLUTS/FLUTS), the International Index of Erectile Function (IIEF), and the Female Sexual Function Index (FSFI) [28-31]
6. Complications including additional surgery, infection, reduced range of motion, and other adverse events
7. Costs and health resource utilization.

## Methods/design

### Overview of study design

FIRST is an ongoing multicenter, blinded RCT of 220 patients who have been diagnosed with FAI and are selected for surgical intervention. Patients are recruited from experienced hip surgeons practicing at the multiple participating sites based both nationally and internationally. Study personnel monitor critical aspects of perioperative care and rehabilitation. We measure pain, function, health-related quality of life, health utility, cost-effectiveness, and will independently adjudicate revision surgeries and other complications over 12 months. Methods Centre ethics approval was obtained from the Hamilton Integrated Research Ethics Board (REB #12-396).

### Patient selection

#### Eligibility criteria

We have broad inclusion criteria in place to improve the feasibility of this trial, as well as patient compliance. All excluded patients will be logged for verification of the generalizability of the results. The inclusion criteria are: 1) adult men or women ages 18 to 50 years, 2) hip pain for greater than 6 months with no relief from non-operative means (physiotherapy, non-steroidal anti-inflammatory medication, rest), 3) documentation of failed physiotherapy, including core conditioning of the hip, back, and abdomen, 4) CAM or Mixed Type FAI as diagnosed on x-rays and magnetic resonance imaging (MRI) or magnetic resonance arthrogram (MRA), 5) temporary relief from an intra-articular hip injection, 6) provision of informed consent from the participant, and 7) ability of the participant to speak, understand and read in the language of the clinical site.

The exclusion criteria are: 1) previous inclusion of the participant in a study involving FAI, 2) evidence of hip dysplasia (centre edge angle less than 20), 3) presence of advanced hip osteoarthritis (Tonnis Grade 2 or 3) [32], 4) presence of other hip syndromes (concurrent non-FAI related pathology), 5) previous trauma to the affected hip, 6) previous surgery on the affected hip or contralateral hip, 7) severe acetabular deformities (e.g. acetabular protrusion, coxa profunda, circumferential labral ossification) [33], 8) immunosuppressive medication use, 9) chronic pain syndromes, 10) significant medical co-morbidities (requiring daily assistance for activities of daily living; ADLs), 11) history of paediatric hip disease (Legg-Calve-Perthes; slipped capital femoral epiphysis), 12) ongoing litigation or compensation claims secondary to hip problems, and 13) any other reasons given, in the surgeon’s judgement, to exclude the patient.

#### Patient recruitment and screening

The FIRST pilot study recruited 50 patients in one year at two centres in Canada and Finland, providing feasibility data and allowing for greater precision for estimates of recruitment and the definitive sample size calculation. We are planning to expand to 10 sites across Canada, Finland, Denmark, and the USA. The first definitive trial patient was randomized on 23 October 2014 Enrollment is ongoing at the time of publication and is expected to be completed by December 2017. All patients presenting to participating surgeons with diagnosed FAI amenable to arthroscopic surgery are screened for participation in the FIRST trial. Such patients are classified as: 1) excluded (if they do not meet the eligibility criteria); 2) missed (presumed eligible but missed due to error or staff availability); or 3) included (eligible and randomized). Study personnel obtain informed consent from all eligible patients.

### Randomization

We use a centralized 24 hour computerized randomization system that allows for automated internet based randomization to allocate patients to the control or intervention group in random block sizes of 4 and 8 prior to surgery. Using this randomization system ensures concealment of treatment allocation [34]. We stratify patients based on centre and impingement sub-type (CAM or Mixed).

### Study interventions

#### Osteochondroplasty

Patients in the intervention group (osteochondroplasty with/without labral repair) have an initial hip evaluation using hip arthroscopy. Three standard hip arthroscopy portals (antero-lateral, mid anterior, distal antero-lateral) are used during the entire procedure to assess and treat the patient. After establishing standard portals, an inter-portal capsulotomy is completed to allow for complete evaluation of the central compartment of the hip. In the central compartment, significant and obvious labral tears and cartilage damage are addressed (repair or debridement). The labrum is repaired if mechanically unstable once probed with visible displacement or chondrolabral separation. The acetabular rim is evaluated and any evident Pincer lesion is resected using an arthroscopic burr under fluoroscopic guidance. Following this resection, the labrum may be re-fixated only if the criteria for labral instability is met. Following this, a limited capsulotomy is completed along the head-neck junction of the femoral neck to allow for visualization and treatment of the impingement lesion in the peripheral compartment. Intraoperative fluoroscopy is used once more to guide the osteochondroplasty and resection of the impingement lesions [21,22,35].

#### Arthroscopic lavage (Control)

Patients in the control group (lavage) have the same three hip portals with limited capsulotomy allowing for a complete assessment of the central and peripheral compartments. The participant has a diagnostic arthroscopy and lavage of the hip joint with three litres of normal saline. No osteochondroplasty or rim resection is completed in the control group. No instruments are used to treat minor cartilage or labral damage. The labrum is only repaired if mechanically unstable once probed with visible displacement or chondrolabral separation. The labrum may be re-fixated only if the criteria for labral instability is met.

During the FIRST pilot phase, of 22 patients randomized to the control arm, only one patient required a large amount (>5 cm^3^) of labral debridement, providing reassurance of general compliance with the control arm protocol.

#### Standardization of postoperative care

Postoperatively, we standardize pain management, protected weightbearing, venous thromboprophylaxis, and physiotherapy. Any deviations from the standardized protocol are documented by the blinded outcome assessors.

#### Surgeon expertise and standardization of procedures

We expect all surgeons involved to have completed at least 30 hip arthroscopic cases, as our prior systematic review suggests that is a critical number of cases to meet a learning curve and decrease complications [14]. Participating surgeons have a standardized video and laboratory demonstration available to them to standardize the surgical techniques for all participants [36]. In addition, all investigators are provided with a standardized postoperative care protocol [37].

### Study outcomes

#### Primary outcome

The primary outcome is the change in pain scores between intervention and control patients at 12 months, as rated using a Visual Analog Scale (VAS) [22,23].

The **VAS** is one of the most frequently used pain rating scales in clinical practice and research [21]. The VAS is a validated unidimensional scale that is easy to use, requires no verbal or reading skills, and is sufficiently versatile to be employed in a variety of settings [38-40].

#### Secondary outcomes

Secondary outcomes include the change in functional outcome, health utility, and quality of life scores using self-administered and interview-administered questionnaires. Questionnaires include a generic health status measurement instrument (SF-12), hip function questionnaires (HOS, iHOT-12), a health utility measure (EQ-5D), and urinary (ICIQ-MLUTS/FLUTS) and sexual function questionnaires (IIEF/FSFI) [26,28-31,41-45]. We will also collect patient cost data, report differences in complication and revision surgery rates, as well as secondary procedures such as anti-inflammatory hip injections.

The **SF-12** may be self or interview-administered and will help document general health status as well as the burden of illness that FAI presents [25]. The **HOS** is a self-administered hip score that was designed to capture hip function and outcomes following surgical therapies such as arthroscopy [24]. The HOS has been shown to have the greatest clinimetric evidence for use in patients with FAI or labral tears [46,47]. The **iHOT-12** is a shorter version of the iHOT-33 designed to be easier to complete in routine clinical practice to measure both health-related quality of life and changes after treatment in young, active patients with hip disorders [26]. This questionnaire has been shown to be valid, reliable, and responsive to change [26]. The **EQ-5D** is a standardized instrument for use as a measure of health outcome [27]. The EQ-5D comprises five dimensions of health (mobility, self-care, usual activities, pain/discomfort, and anxiety/depression). The EQ-5D has been used in previous studies involving patients with hip pain and has been extensively validated [48,49]. Our decision to the use EQ-5D was based upon our interest in collecting health utility data for a formal cost-effectiveness analysis.

Because arthroscopy of the hip is more technically challenging than for other joints due to the deep-seated nature of the hip joint, the surrounding soft tissue envelope, and the encapsulated ball and socket configuration, any excessive, inadequate or improperly applied traction could result in compression injury to the perineum, pudendal neuropraxia, and skin complications, sometimes causing urinary and/or sexual dysfunction [50-53]. The two validated questionnaires selected that pertain to male and female urinary symptoms are gender specific variations of the **ICIQ-MLUTS** (male) and **ICIQ-FLUTS** (female). These are validated patient-completed questionnaires, which evaluate lower urinary tract symptoms (LUTS), as well as quality of life [28,29]. Both questionnaires have demonstrated validity, reliability and responsiveness internally and externally. The **FSFI** is a brief psychometrically sound and reliable tool that assesses female sexual function, and has proven ability to discriminate between clinical and nonclinical populations [30]. The FSFI is also designed to measure the impact of sexual function on quality of life [30]. The **IIEF** is a brief self-administered questionnaire assessing sexual experience within the past 4 weeks, consisting of 15 questions designed to address 5 relevant aspects of male sexual function; specifically erectile function, sexual desire, orgasmic function, intercourse satisfaction and overall satisfaction [31,54]. This instrument is psychometrically sound with high sensitivity and specificity and has been validated for administration in research and clinical settings across cultures with linguistically validated versions [31,54].

The questionnaires are validated for many of the countries where our trial centres are located. All questionnaires will be translated to the site-specific primary language. We will record all changes in pain medication as well as any hip-specific complications and adverse events (Table 1). We will also conduct a cost analysis based on collected cost data (using a patient cost diary) and the health utility questionnaire.

**Table 1:** **FIRST schedule of events**

**Assessment**	**Screening**	**Enrolment (Baseline)**	**Surgery**	**2 Weeks**	**6 Weeks**	**3 Months**	**6 Months**	**12 Months**
Screening form	**X**							
Informed consent	**X**							
Randomization form		**X**						
Baseline characteristics form		**X**						
Hip characteristics form		**X**						
Surgical form			**X**					
Arthroscopic findings form			**X**					
Perioperative form			**X**					
Follow-up form				**X**	**X**	**X**	**X**	**X**
Complications form (if required)			**(X)**	**(X)**	**(X)**	**(X)**	**(X)**	**(X)**
Pain medication log (if required)			**(X)**	**(X)**	**(X)**	**(X)**	**(X)**	**(X)**
X-rays and/or MRI	**X**			**X**				**X**
Pain Visual Analog Scale (VAS)		**X**		**X**	**X**	**X**	**X**	**X**
Hip Outcome Score (HOS)		**X**		**X**	**X**	**X**	**X**	**X**
Short Form 12 (SF-12)		**X**		**X**	**X**	**X**	**X**	**X**
International Hip Outcome Tool (iHOT-12)		**X**		**X**	**X**	**X**	**X**	**X**
EuroQol 5D (EQ-5D)		**X**		**X**	**X**	**X**	**X**	**X**
International Consultation on Incontinence Questionnaire Male/Female Lower Urinary Tract Symptoms (ICIQ-MLUTS/FLUTS)		**X**			**X**			**X**
International Index of Erectile Function (Male) (IIEF)		**X**			**X**			**X**
Female Sexual Function Index (FSFI)		**X**			**X**			**X**

### Study follow-up

**Figure 2:**
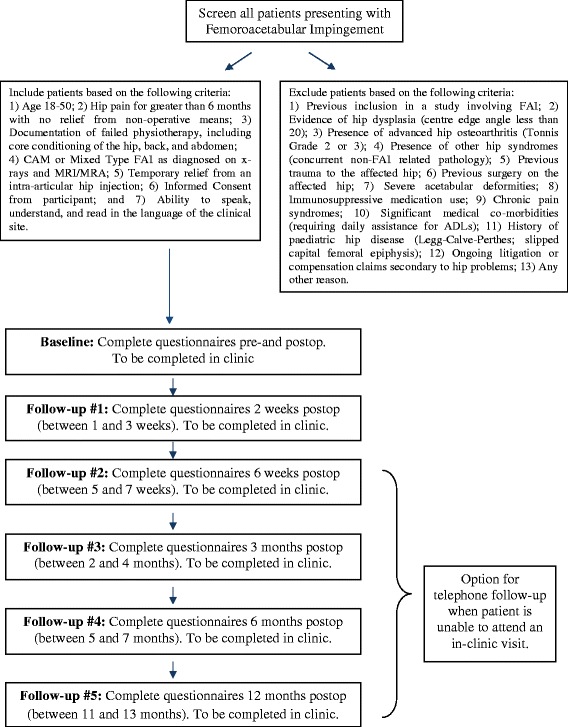
**FIRST process overview.**

Patients undergo baseline evaluation with regular follow-up visits at 2 weeks, 6 weeks, 3 months, 6 months, and 12 months postoperatively. This follow up schedule is in accordance with the current practice at each trial centre and does not require extra visits or costs to the patients. Patients who are unable to attend the follow-up appointments will be contacted by phone to complete the questionnaires. The full study process is shown in Figure 2.

Our decision to follow patients to one year represents a practical and trial cost compromise between earlier perioperative outcomes and longer term sequelae of FAI (5 years or more). The current literature is limited by the reporting of short term outcomes with follow-up ranging from 6 months to 5.2 years [55]. Outcomes between one year and 5 years have been shown to be not significantly different [56,57]. Further, we surveyed experts in FAI surgery in North America and identified that most surgeons expect maximum improvement by 12 months following surgery [20]. Thus, it is unlikely that follow-up beyond one year will yield a differential treatment effect from FAI surgery versus controls, and the results will remain clinically meaningful.

### Protecting against sources of bias

We have implemented multiple methods for protecting against bias. We conceal randomization, maximize possible blinding, utilize strategies to limit loss to follow-up and crossovers, and objectively adjudicate patient outcomes.

#### Blinding

Patients, outcomes assessors and data analysts are blinded to patient allocation by limiting their access to surgical records and postoperative x-rays. Blinding of patients is feasible because their postoperative care (physiotherapy etc.) does not differ between treatment groups. To participate in the trial, patients provide consent that limits their access to postoperative x-rays or surgical notes until the follow-up period for the trial is complete.

#### Maximizing patient follow-up

We have implemented several procedures to limit loss of follow-up, including excluding individuals who are likely to present problems with follow-up, obtaining extensive contact information from each consented patient, having local study personnel remind patients of upcoming clinic visits, and ensuring that follow-up visits coincide with normal surgical clinic visits (Table 2). Using these strategies during the pilot study, we were able to achieve a 95% follow-up rate.

**Table 2:** **FIRST enrolment and follow-up enhancement strategies**

**Before the study begins**	**At screening**	**At baseline**	**During the study**	**For participants who are difficult to contact**
• Formally train clinical research coordinators on how to reduce loss to follow-up	• Let participants know what information will be collected and how their information will be used	• Obtain several personal contacts on a locator form, including friend or family and employment contacts to assist in locating participants later	• Provide participant with a choice of email, phone, and/or clinic visits for convenience	• Repeatedly search for updated information or try previously disconnected phone numbers
• Ensure the survey area is private and comfortable	• Let participants know that the study might help other patients in similar situations to enhance their motivation to participate	• Ensure each locator form is signed by the participant to ensure a thorough understanding and to give written consent to contact listed individuals	• Be flexible on scheduling in-clinic visits to allow for scheduling issues to be resolved	• Search local phone directories, contact alternate contacts, try to contact patients from a different phone number or at a different time of day
• Develop a locator protocol to guide efforts to locate patients for follow-up visits	• Be explicit with the participant about the follow-up procedures including providing specific information to the participant about when to expect contact from the study staff, how often, and what type of contact (email, in-person, telephone, etc.)	• Ensure informed consent is conducted in an appropriate manner, including using examples and explanations that are accessible to a lay audience	• Ensure participants can easily contact the study staff by providing them with materials on which the study toll-free number was printed, such as business cards, appointments cards, brochures, or study promotional materials	• Hold regular staff meetings to brainstorm ideas about how to find some of the participants who are the most difficult. This will also increase staff motivation for locating hard-to-find participants
			• Routinely verify contact information	
			• Study personnel will log all attempts to contact each participant and the outcome of each attempt	
			• The methods centre will conduct random site audits to verify that all precautions are being taken to secure data	
			• The methods centre will conduct extensive data entry audits and verifications, weekly data reports, recruitment reports, and contact tracking reports	

#### Minimizing crossovers

Crossovers are extremely unlikely between the osteochondroplasty and lavage groups because both involve arthroscopic approaches and the same surgeon will be performing all procedures at each site. Any patients who do crossover will be analyzed in the group to which they were randomized, maintaining the intention to treat approach we plan to use for the analyses. There were no crossovers for the first 50 patients in the pilot phase of the trial, supporting our assertion that crossovers will be rare.

#### Adjudication

An independent, blinded Adjudication Committee will review patient eligibility (e.g. preoperative radiographic alpha angle [15,58,59]), intraoperative arthroscopic findings, and all reported complications. The committee is comprised of three orthopaedic surgeons with expertise in hip surgery and adjudication. All centres submit x-rays and/or MRI images and relevant hospital records to be included in the adjudication process. Any disagreements between the Adjudication Committee members will be resolved during regular conference calls. If a consensus cannot initially be reached, additional information will be requested from the participating site to clarify areas of uncertainty.

### Sample size calculation

We powered the FIRST trial to detect a minimal clinically important improvement (MCII) in the VAS pain score (improvement of at least 13 points) between hip osteochondroplasty and lavage. The estimates of MCII were based upon Norman et al. and estimates from our pilot clinical trial [60]. To achieve 80% study power and using at two-sided Type I error rate (5%), our trial requires 73 patients per study arm.

For the secondary outcomes, we set the two-tailed Type I error rate to 1% to account for multiple comparisons. We consider an important difference in the SF-12 to correspond to a moderate effect as reported by Cohen [61], as well as a minimally important difference (MID) in the SF-12 as reported by Ware [62]. In both cases, the value is at least half the standard deviation, equivalent to a 4-point difference in score. Specifying an alpha level of 0.01 and a beta of 0.20 (study power = 0.80), we require a sample of at least 192 patients (96 per group) to ensure detection of a half standard deviation in improvement. The HOS, under the assumption of a MID of 13 points, will require 93 patients per treatment group. The iHOT-12 will require 85 patients per group given the MID of 6.1 from Mohtadi et al.[47].

Therefore, for adequate study power across all our planned outcome measures, we will need to recruit and follow 192 patients. To account for potential loss to follow up (5%) and potential crossovers (5%), FIRST will recruit 107 patients per treatment arm, rounded to 220 patients in total.

### Statistical plan

#### Primary analyses

We will adopt the intention to treat principle for all analyses—that is, patients will be retained in the groups to which they were randomized. The baseline characteristics of the patients will be summarized by group, reported as a mean (standard deviation) or median (first quartile, third quartile) for continuous variables and count (percent) for categorical variables. We will use an analysis of covariance (ANCOVA) to compare the mean pain scores (VAS) at 12 months post-surgery adjusting for baseline scores. The treatment effect will be quantified with an absolute difference in rate of pain reduction with the associated 95% confidence interval (CI) and p-value. All p-values will be reported to 3 decimal places with those less than 0.001 reported as p < 0.001. The criterion for statistical significance will be set at alpha = 0.05. Multiple regression models will be used to determine variables and factors related to improvement in pain and quality of life scores.

#### Secondary analyses

We will estimate the effect of arthroscopic osteochondroplasty (intervention) versus lavage (control) on FAI patient quality of life (SF-12), function (HOS, iHOT-12), health outcome (EQ-5D), and sexual/urinary function (ICIQ-MLUTS/FLUTS, FSFI, IIEF) at 12 months with ANCOVA using the following covariates: 1) baseline scores and 2) impingement sub-type. We will use multiple imputation to handle missing data to enable an intention to treat analysis [63]. The results will be reported as means with 95% CIs. We will use the Bonferroni method to adjust the p-value for multiple secondary outcomes.

#### Sensitivity and subgroup analyses

We will perform the following sensitivity analyses: 1) *centre-effects:* we will redo both primary and secondary analyses adjusting for centre as fixed and random-effects; 2) *per-protocol analysis:* we will also redo the analyses including patients who received the interventions as allocated; and 3) a*djusted analyses:* we will perform adjusted analyses to address any residual baseline imbalance between groups [64].

We plan to conduct a subgroup analysis comparing the treatment effects in patients with severe (alpha angle greater than 83 degrees), moderate (alpha greater than 60 degrees), and mild (alpha angle of less than 60 degrees) impingement at baseline. Surgeons often describe FAI deformity in ranges from mild, moderate, to severe [65]. As such, an analysis of outcomes based on the severity of the deformity would be informative to both patients and surgeons. We plan to use ANCOVA models and include treatment by subgroup interactions to assess whether the magnitude of the treatment effect is significantly different between subgroups [7,65,66].

### Data management

The Case Report Forms (CRFs) are the primary data collection tool for the study. An Electronic Data Capture system (iDataFax) is being used to submit data to the Methods Centre located at McMaster University. Upon receipt of the data, the personnel at the Methods Centre make a visual check of the data and query all missing, implausible, and inconsistent data.

### Data safety and monitoring committee

The purpose of the Data Safety and Monitoring Committee (DSMC) is to advise the FIRST Investigators regarding the continuing safety of the trial participants. The DSMC is comprised of a clinical expert with prior trial experience, a clinical trial methodologist, and a biostatistician. All members are independent of the trial investigators, and have neither financial nor scientific conflicts of interest with the trial.

### Ethical considerations

All patients included in FIRST will sign a site-specific, Ethics Board-approved consent form that describes this study and provides sufficient information for patients to make an informed decision about their participation. All participating centres must obtain Ethics Board approval from their institution for the study protocol, the consent form template, the CRFs, and any additional protocol amendments. Any protocol amendments will be communicated to the site investigators, the Ethics Board, trial participants, and trial registries as necessary.

Information about study patients will be kept confidential and will be managed in accordance with the following rules: 1) All study-related information is stored securely at the clinical site, 2) All study patient information is stored in locked file cabinets and is accessible only to study personnel, 3) All CRFs are identified only by a coded patient number and initials, 4) All records that contain patient names, or other identifying information, are stored separately from the study records that are identified only by the coded patient number and initials, and 5) All local databases are password protected.

## Discussion

The rationale for the FIRST trial includes: 1) a growth in popularity during the last 10 years in the surgical management of FAI; 2) global uncertainty in the surgical community regarding efficacy of surgical management of FAI; 3) a lack of compelling RCTs evaluating the efficacy and safety of FAI surgery on patient important outcomes; 4) a large body of preparatory research including systematic reviews, surveys, and agreement studies; and 5) growing international support and feasibility for a definitive surgical trial addressing FAI based upon our highly successful pilot phase (n = 50 patients).

The FIRST trial is one of the first RCTs to evaluate the potential benefits in outcome purported to arise from the surgical management of FAI. This trial will overcome many of the limitations and associated biases of the current literature. The trial sample size (N = 220) was calculated based on pilot study data to ensure there will be sufficient statistical power to detect differences across several patient important outcomes, including pain, function, and quality of life. Our pilot has also demonstrated feasibility through our ability to recruit patients efficiently; investigator compliance with key aspects of the protocol; maintenance of data quality; maintenance of high follow-up rates; and the research team’s ability to organize and coordinate trial procedures in a multinational trial. Furthermore, the FIRST trial has methodological safeguards including the use of a centralized system to randomize patients; blinding patients, outcome assessors, data analysts, and the Steering Committee; standardization and documentation of peri-operative care; the use of strategies to limit loss to follow up; and adjudication of trial events by an independent Adjudication Committee.

As with any surgical RCT, a limitation of the FIRST trial is that surgeons cannot be blinded to the treatment allocation. Given that FAI is a relatively newly diagnosed condition, there are few expert surgeons that identify and treat it, and many have differing opinions on the ideal surgical approach and the amount of soft tissue repair required for these patients. We have put protocols in place to limit possible inconsistencies across sites by standardizing and monitoring critical aspects of operative and peri-operative care, including preclusion of extensive repair in patients allocated to the control group (unless deemed necessary by the participating surgeon), pain management, protected weight bearing, venous thromboembolism thromboprophylaxis, and physiotherapy. We will also only invite surgeons who are experts in FAI surgery to participate in this trial.

The 5-fold increase in literature promoting the surgical management of FAI (2005–2010) has focused on techniques and early outcomes, predominantly through case series lacking controls and bias reducing measures [16]. It remains plausible that, in the absence of high quality comparative evidence, the increase in FAI procedures, largely driven by observational studies, may result in unnecessary surgery, potential harm, and increased health care costs [11]. With an estimated FAI prevalence of 14-17% in the general North American population (at least 74 million persons), optimizing treatment has the potential to improve the lives of millions of young, active persons who are diagnosed with hip impingement [1,2]. Improving outcomes will directly reduce the economic burden of FAI. Few orthopaedic surgical trials have similar potential to shift the paradigm of care dramatically towards (or away) from surgical bony and soft tissue interventions, likely leading to changes in sports medicine practice.

## Abbreviations

ANCOVA: Analysis of Covariance
CI: Confidence Interval
CRF: Case Report Form
DSMC: Data Safety and Monitoring Committee
EQ-5D: EuroQol 5 Dimensions
FAI: Femoroacetabular Impingement
FIRST: Femoroacetabular Impingement RandomiSed controlled Trial
FSFI: Female Sexual Function Index
HOS: Hip Outcome Score
ICIQ-FLUTS: International Consultation on Incontinence Questionnaire Female Lower Urinary Tract Symptoms
ICIQ-MLUTS: International Consultation on Incontinence Questionnaire Male Lower UrinaryTract Symptoms
IIEF: International Index of Erectile Function
iHOT-12: International Hip Outcome Tool
MCII: Minimal Clinically Important Improvement
MID: Minimally Important Difference
MRA: Magnetic Resonance Arthrogram
MRI: Magnetic Resonance Imaging
RCT: Randomized controlled trial
SF-12: Short Form-12
VAS: Visual Analog Scale

## References

[1] Gosvig KK, Jacobsen S, Sonne-Holm S, Gebuhr P (2008). The prevalence of cam-type deformity of the hip joint: a survey of 4151 subjects of the Copenhagen Osteoarthritis Study. Acta Radiol.

[2] Hack K, Di Primio G, Rakhra K, Beaulé PE (2010). Prevalence of cam-type femoroacetabular impingement morphology in asymptomatic volunteers. J Bone Joint Surg Am.

[3] Kapron AL, Anderson AE, Aoki SK, Phillips LG, Petron DJ, Toth R (2011). Radiographic prevalence of femoroacetabular impingement in collegiate football players: AAOS Exhibit Selection. J Bone Joint Surg Am.

[4] Beck M, Kalhor M, Leunig M, Ganz R (2005). Hip morphology influences the pattern of damage to the acetabular cartilage: femoroacetabular impingement as a cause of early osteoarthritis of the hip. J Bone Joint Surg (Br).

[5] Ganz R, Parvizi J, Beck M, Leunig M, Nötzli H, Siebenrock KA (2003). Femoroacetabular impingement: a cause for osteoarthritis of the hip. Clin Orthop Relat Res.

[6] Byrd JW, Jones KS (2011). Arthroscopic management of femoroacetabular impingement: minimum 2-year follow-up. Arthroscopy.

[7] Agricola R, Heijboer MP, Bierma-Zeinstra SM, Verhaar JA, Weinans H, Waarsing JH (2013). CAM impingement causes osteoarthritis of the hip: a nationwide prospective cohort study (CHECK). Ann Rheum Dis.

[8] Clohisy JC, Dobson MA, Robison JF, Warth LC, Zheng J, Liu SS (2011). Radiographic structural abnormalities associated with premature, natural hip-joint failure. J Bone Joint Surg Am.

[9] Colvin AC, Harrast J, Harner C (2012). Trends in hip arthroscopy. J Bone Joint Surg Am.

[10] Bozic KJ, Chan V, Valone FH, Feeley BT, Vail TP (2013). Trends in hip arthroscopy utilization in the United States. J Arthroplasty.

[11] Millennium Research Group. An evolution in keyhole surgery: arthroscopy goes for the hip. IQ Industry Insight. 2009, 1–3. Ref Type: Pamphlet.

[12] Kolata G. Hip procedure grows popular despite doubt. The New York Times. 15 Nov 2011. [http://www.nytimes.com/2011/11/16/health/hip-impingement-grows-popular-but-remains-unproven.html?_r=0].

[13] Clohisy JC, Knaus ER, Hunt DM, Lesher JM, Harris-Hayes M, Prather H (2009). Clinical presentation of patients with symptomatic anterior hip impingement. Clin Orthop Relat Res.

[14] Hoppe DJ, de Sa D, Simunovic N, Bhandari M, Safran MR, Larson CM (2014). The learning curve for hip arthroscopy: a systematic review. Arthroscopy.

[15] de Sa D, Urquhart N, Philippon M, Ye JE, Simunovic N, Ayeni OR (2014). Alpha angle correction in femoroacetabular impingement. Knee Surg Sports Traumatol Arthrosc.

[16] Hetaimish BM, Khan M, Crouch S, Simunovic N, Bedi A, Mohtadi N (2013). Consistency of reported outcomes after arthroscopic management of femoroacetabular impingement. Arthroscopy.

[17] Ayeni OR, Chan K, Al-Asiri J, Chien T, Sprague S, Liew S (2013). Sources and quality of literature addressing femoroacetabular impingement. Knee Surg Sports Traumatol Arthrosc.

[18] Ayeni OR, Naudie D, Crouch S, Adili A, Pindiprolu B, Chien T (2013). Surgical indications for treatment for femoroacetabular impingement with surgical hip dislocation. Knee Surg Sports Traumatol Arthrosc.

[19] Ayeni OR, Adamich J, Farrokhyar F, Simunovic N, Crouch S, Philippon MJ (2014). Surgical management of labral tears during femoroacetabular impingement surgery: a systematic review. Knee Surg Sports Traumatol Arthrosc.

[20] Ayeni OR, Belzile EL, Musahl V, Naudie D, Crouch S, Sprague S (2014). Results of the PeRception of femOroaCetabular impingEment by Surgeons Survey (PROCESS). Knee Surg Sports Traumatol Arthrosc.

[21] Price DD, McGrath PA, Rafii A, Buckingham B (1983). The validation of visual analogue scales as ratio scale measures for chronic and experimental pain. Pain.

[22] Larson CM, Giveans MR (2008). Arthroscopic management of femoroacetabular impingement: early outcomes measures. Arthroscopy.

[23] Larson CM, Giveans MR (2009). Arthroscopic debridement versus refixation of the acetabular labrum associated with femoroacetabular impingement. Arthroscopy.

[24] Schenker ML, Martin R, Weiland DE, Philippon MJ (2005). Current trends in hip arthroscopy: a review of injury diagnosis, techniques and outcome scoring. Curr Opin Orthop.

[25] Ware J, Kosinski M, Keller SD (1996). A 12-item short-form health survey: construction of scales and preliminary tests of reliability and validity. Med Care.

[26] Griffin DR, Parsons N, Mohtadi NGH, Safran MR on behalf of the Multicenter Arthroscopy of the Hip Outcomes Research Network (MAHORN) (2012). A short version of the International Hip Outcome Tool (iHOT-12) for use in routine clinical practice. Arthroscopy.

[27] EuroQol Group. The EQ-5D. [http://www.euroqol.org/home.html].

[28] Jackson S, Donovan J, Brookes S, Eckford S, Swithinbank L, Abrams P (1996). The Bristol Female Lower Urinary Tract Symptoms questionnaire: development and psychometric testing. Br J Urol.

[29] Donovan J, Abrams P, Peters TJ, Kay HE, Reynard J, Chapple C (1996). The ICS-‘BPH’ study: the psychometric validity and reliability of the ICS male questionnaire. Br J Urol.

[30] Rosen R, Brown C, Heiman J, Leiblum S, Meston C, Shabsigh R (2000). The Female Sexual Function Index (FSFI): a multidimensional self-report instrument for the assessment of female sexual function. J Sex Marital Ther.

[31] Rosen RC, Riley A, Wagner G, Osterloh IH, Kirkpatrick J, Mishra A (1997). The International Index of Erectile Function (IIEF): a multidimensional scale for assessment of erectile dysfunction. Urology.

[32] Tonnis D, Heinecke A (1999). Current concepts review-Acetabular and femoral anteversion: relationship with osteoarthritis of the hip. J Bone Joint Surg Am.

[33] Zaltz I, Kelly BT, Larson CM, Leunig M, Bedi A (2014). Surgical treatment of femoroacetabular impingement: what are the limits of hip arthroscopy?. Arthroscopy.

[34] Haynes S (2006). Guyatt and Tugwell: Clinical Epidemiology: How to do Clinical Practice Research.

[35] Kelly BT, Weiland DE, Schenker ML, Philippon MJ (2005). Arthroscopic labral repair in the hip: surgical technique and review of the literature. Arthroscopy.

[36] Ayeni OR, Wong I, Chien T, Musahl V, Kelly BT, Bhandari M (2012). Surgical indications for arthroscopic management of femoroacetabular impingement. Arthroscopy.

[37] Spencer-Gardner L, Eischen JJ, Levy BA, Sierra RJ, Engasser WM, Krych AJ (2014). A comprehensive five-phase rehabilitation programme after hip arthroscopy for femoroacetabular impingement. Knee Surg Sports Traumatol Arthrosc.

[38] Jensen MP, Karoly P (1986). The measurement of clinical pain intensity: a comparison of six methods. Pain.

[39] Collins S, Moore A, McQuay H (1997). The visual analog pain intensity scale: what is moderate pain in millimeters?. Pain.

[40] Ho K, Spence J, Murphy M (1996). Review of pain measurement tools. Ann Emerg Med.

[41] Kemp JL, Collins NJ, Roos EM, Crossley KM (2013). Psychometric properties of patient-reported outcome measures for hip arthroscopic surgery. Am J Sports Med.

[42] Hinman RS, Dobson F, Takla A, O’Donnell J, Bennell KL (2014). Which is the most useful patient-reported outcome in femoroacetabular impingement? Test-retest reliability of six questionnaires. Br J Sports Med.

[43] Martin RL, Kelly BT, Philippon MJ (2006). Evidence of validity for the hip outcome score. Arthroscopy.

[44] Martin RL, Philippon MJ (2008). Evidence of reliability and responsiveness for the hip outcome score. Arthroscopy.

[45] Martin RL, Philippon MJ (2007). Evidence of validity for the hip outcome score in hip arthroscopy. Arthroscopy.

[46] Throborg K, Roos EM, Bartels EM, Petersen J, Hölmich P (2010). Validity, reliability and responsiveness of patient-reported outcome questionnaires when assessing hip and groin disability: a systematic review. Br J Sports Med.

[47] Mohtadi NGH, Griffin DR, Pedersen ME, Chan D, Safran MR, Parsons N (2012). The development and validation of a self-administered quality-of-life outcome measure for young, active patients with symptomatic hip disease: the International Hip Outcome Tool (iHOT-33). Arthroscopy.

[48] Bosch JL, Hunink MG (2000). Comparison of the Health Utilities Index Mark 3 (HUI3) and the EuroQol EQ-5D in patients treated for intermittent claudication. Qual Life Res.

[49] Hurst NP, Kind P, Ruta D, Hunter M, Stubbings A (1997). Measuring health-related quality of life in rheumatoid arthritis: validity, responsiveness and reliability of EuroQol (EQ-5D). Br J Rheumatol.

[50] Byrd JW (1998). Complications associated with hip arthroscopy. Operative Arthroscopy.

[51] Papvasiliou AV, Bardakos NV (2012). Complications of arthroscopic surgery of the hip. Bone Joint Res.

[52] Labat JJ, Riant T, Robert R, Amarenco G, Lefaucheur JP, Rigaud J (2008). Diagnostic criteria for pudendal neuralgia by pudendal nerve entrapment (Nantes criteria). Neurourol Urodyn.

[53] Harris JD, McCormick FM, Abrams GD, Gupta AK, Ellis TJ, Bach BR (2013). Complications and reoperations during and after hip arthroscopy: a systematic review of 92 studies and more than 6000 patients. Arthroscopy.

[54] Sarramon JP, Malavaud B, Braud F, Bertrand N, Vaessen C, Rischmann P (2001). Evaluation of male sexual function after deep dorsal vein arterialization of the penis. J Urology.

[55] Ng VY, Arora N, Best TM, Pan X, Ellis TJ (2010). Efficacy of surgery for femoroacetabular impingement: a systematic review. Am J Sports Med.

[56] Malviya A, Stafford GH, Villar RN (2012). Impact of arthroscopy of the hip for femoroacetabular impingement on quality of life at a mean follow-up of 3.2 years. J Bone Joint Surg (Br).

[57] Philippon MJ, Ejnisman L, Ellis HB, Briggs KK (2012). Outcomes 2 to 5 years following hip arthroscopy for femoroacetabular impingement in the patient aged 11 to 16 years. Arthroscopy.

[58] Nötzli HP, Wyss TF, Stoecklin CH, Schmid MR, Treiber K, Hodler J (2002). The contour of the femoral head-neck junction as a predictor for the risk of anterior impingement. J Bone Joint Surg (Br).

[59] Clohisy JC, Carlisle JC, Beaulé PE, Kim YJ, Trousdale RT, Sierra RJ (2008). A systematic approach to the plain radiographic evaluation of the young adult hip. J Bone Joint Surg Am.

[60] Norman GR, Sloan JA, Wyrwich KW (2003). Interpretation of changes in health-related quality of life: the remarkable universality of half a standard deviation. Med Care.

[61] Cohen J (1988). Statistical Power Analysis for the Behavioral Sciences.

[62] Ware JE, Sherbourne CD (1992). The MOS 36-item short form health survey (SF-36). I. Conceptual framework and item selection. Med Care.

[63] Little RJA, Rubin DB (1987). Statistical Analysis with Missing Data.

[64] Thabane L, Mbuagbaw L, Zhang S, Samaan Z, Marcucci M, Ye C (2013). A tutorial on sensitivity analyses in clinical trials: the what, why, when and how. BMC Med Res Methodol.

[65] Agricola R, Waarsing JH, Thomas GE, Carr AJ, Reijman M, Bierma-Zeinstra SM (2014). CAM impingement: defining the presence of a CAM deformity by the alpha angle: data from the CHECK cohort and Chingford cohort. Osteoarthritis Cartilage.

[66] Agricola R, Waarsing JH, Arden NK, Carr AJ, Bierma-Zeinstra SM, Thomas GE (2013). CAM impingement of the hip-a risk factor for hip osteoarthritis. Nat Rev Rheumatol.

